# Impact of Soil Heavy Metal Pollution on Food Safety in China

**DOI:** 10.1371/journal.pone.0135182

**Published:** 2015-08-07

**Authors:** Xiuying Zhang, Taiyang Zhong, Lei Liu, Xiaoying Ouyang

**Affiliations:** 1 Jiangsu Provincial Key Laboratory of Geographic Information Science and Technology, International Institute for Earth System Science, Nanjing University, Nanjing, 210023, China; 2 School of Geographic and Oceanographic Sciences, Nanjing University, Nanjing, 210023, China; 3 Jiangsu Center for Collaborative Innovation in Geographical Information Resource Development and Application, Nanjing, 210023, China; 4 State Key Laboratory of Remote Sensing Science, Institute of Remote Sensing and Digital Earth, Chinese Academy of Sciences, Beijing, 100101, China; China National Rice Research Institute, CHINA

## Abstract

Food safety is a major concern for the Chinese public. This study collected 465 published papers on heavy metal pollution rates (the ratio of the samples exceeding the Grade II limits for Chinese soils, the Soil Environmental Quality Standard-1995) in farmland soil throughout China. The results showed that Cd had the highest pollution rate of 7.75%, followed by Hg, Cu, Ni and Zn, Pb and Cr had the lowest pollution rates at lower than 1%. The total pollution rate in Chinese farmland soil was 10.18%, mainly from Cd, Hg, Cu, and Ni. The human activities of mining and smelting, industry, irrigation by sewage, urban development, and fertilizer application released certain amounts of heavy metals into soil, which resulted in the farmland soil being polluted. Considering the spatial variations of grain production, about 13.86% of grain production was affected due to the heavy metal pollution in farmland soil. These results many provide valuable information for agricultural soil management and protection in China.

## Introduction

Heavy metals with adverse health effects in human metabolism (including lead (Pb), cadmium(Cd), mercury(Hg), Arsenic(As), Copper (Cu), Nickel (Ni), Zinc(Zn), and chromium (Cr)) present obvious concerns due to their persistence in the environment and documented potential for serious health consequences. These toxic substances could reach human beings through various absorption pathways of ingestion, dermal contact, diet through the soil-food chain, inhalation, and oral intake [1–3]. In a soil-vegetable or soil-grain system, diet dominated the exposure pathways of heavy metals to humans [4,5]. Soil is an important source for heavy metals in crops and vegetables since the plants’ roots can absorb these pollutants from soil, and transfer them to seeds [6]. In addition to the rapid reduction of arable land, soil contamination by heavy metals poses an often overlooked but no less critical threat to China's food security[7,8].

In 1995, the Ministry of Environmental Protection (MEP) of China issued the Soil Environmental Quality Standard (GB 15618–1995), which was developed through large numbers of experiments and soil surveys[9].It provides the allowed concentration limit of soil pollutants and monitoring methods, considering soil functions, protection objectives and soil parameters of pH and CEC(Cation Exchange Capacty). The aim of this standard is to prevent soil pollution, and protect soil function, eco-environment, agricultural & forestry production and human health. There are three grades for the soils, among them the Grade II limits of heavy metals are used for farmland soil to guarantee healthy plant growth and safe grain productions. If the concentrations of heavy metal are higher than the limits sets in Grade II, the soil samples are considered polluted[10]. This standard has been one of the most important legal bases and criteria for soil quality protection and pollution prevention in China[11].

With rapid industrialization and the wide application of agrochemicals in agricultural activities in recent years, China is now facing great challenges in heavy metal contamination in farmland soil [4,12,13]. Although lots of studies on heavy metal pollutions focused on local areas in China, little is known on a national scale. The national survey of soils from 2005 to 2013 investigated about 6.3×10^6^ km^2^ of land throughout China and showed that 19.40% of the investigated farmland soil samples were polluted, and the main pollutants were heavy metals (Bulletin of National Soil Pollution Survey, 2014). Furthermore, a study by Song et al. (2013) reviewed 127 studies and reported that the serious heavy metal pollutions in farmland soil were caused by Cd, Ni, Hg, As, and Pb; while the pollution rates of Zn, Cr, and Cu were low. Another study investigated 131 soil samples from farmland throughout China and gave the concentration levels of heavy metals, showing that the 6.11%, 1.53%, 9.16%, and 6.11% of the samples exceeded the less stringent grade II limits for Cd, Zn, Cu, and Ni, respectively, and no sample exceeded the standard for Pb and Cr[14]. Therefore, we still do not know the spatial distribution of the affected areas by heavy metals, and have no idea of their influences on food safety throughout China.

To prevent further heavy metal pollution in soil and enact effective measures to remediate metal contamination, it is essential to understand the contaminated areas and their sources on a national scale. This study tried to resolve the afore-mentioned problems, based on the data collected from the studies of published papers. The present study aims to: (1) identify the potential sources of heavy metal in farmland soil, (2) obtain the spatial distribution of affected farmland soil by heavy metals, and (3) evaluate the influences of heavy metal on food security.

## Data Collection and Methods

### Data collection

**Fig 1:**
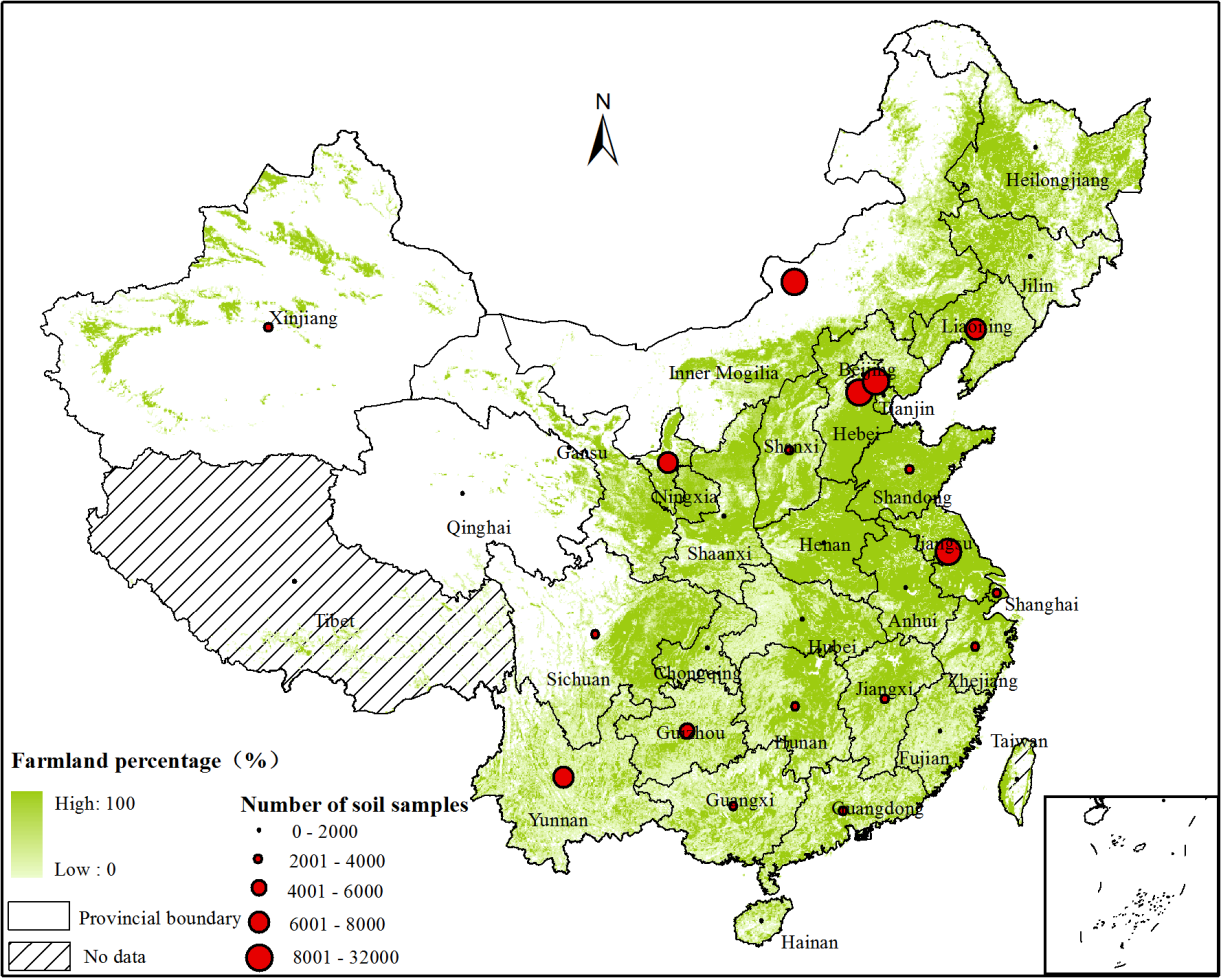
Number of the soil samples in the collected data records

In this study, the pollution rates of heavy metal in topsoil (0–20 or 0–15 cm) of farmland throughout China are collected from the studies of published papers from 2005 to 2014. The selection process of the relevant papers has been described in the study by Zhang[15]. In total, 612 data records of heavy metal pollution rates in 465 peer-reviewed articles are collected. These data are evaluated based on the Soil Environmental Quality Standard -1995, ranging from 0 to 100%. They are calculated by the number of soil samples higher than the reference II divided by the total number of investigated soil samples. Moreover, the basic information such as the location of the study area, potential sources, number of soil samples, heavy metal pollution rate, references, is obtained from each study (S1 Table). The number of soil samples in the collected data records in each province is described in Fig 1.

The land category information of the collected data records including the areas around a) non-ferrous metal mining and smelting activities, b) non-metallic mining and smelting activities, c) industrial activities, d) irrigation area, e) urban and peri-urban area, and f) remote area (the area without obvious point pollution sources) is collected from the original studies. Heavy metal pollution rates are separately recorded to study the contribution of potential sources for heavy metal pollution in arable soil.

### Pollution rates of individual heavy metal

The sample numbers in each study are used as the weights when calculating the average of the pollution rate of the heavy metals due to the considerable variations of sample numbers in each data record. This means that the study with more soil samples would give a greater contribution to the pollution rates at a regional or national scale. The sample-number-weighted mean(*SPR*_*j*_) of the pollution rate of the heavy metal *j* is calculated as:

$$SPR_{j}=\frac{R_{i,j}\times N_{i,j}}{\sum_{i=1}^{n}N_{i,j}}$$ (1)

where *i* is *the data record i*, *j* is the heavy metal *j*, *N*_*i*,*j*_ is the sampling number in the data record *i* for the heavy metal *j*, *R*_*i*,*j*_ is the pollution rates of the heavy metal *j* in the data record *i*. *N*_*i*,*j*_ and *R*_*i*,*j*_ are obtained from the original studies.

### Pollution rate of heavy metals in farmland soil on a regional scale

We aim to evaluate the influence of heavy metal pollution on food safety, thus the maximum pollution rate among the investigated heavy metals in each data record is used to denote the pollution situation in the study area. The heavy metal pollution rates on a provincial scale (*RSPR*_*m*_) is calculated:

$$RSPR_{m}=\frac{MAXR_{i,m}\times N_{i,m}}{\sum_{i=1}^{n}N_{i,m}}$$ (2)

where *i* is *the data record i*, *m* is the province *m*, *N*_*i*,*m*_ is the sampling number in the data record *i* in the province *m*, MAX*R*_*i*,*m*_ is the pollution rate of the heavy metal in province *m*. *N*_*i*,*m*_ and *R*_*i*,*m*_ are obtained from the original studies. When calculating the pollution rate of heavy metals in Chinese farmland soil, *m* refers to the coverage of China.

### Assessment of soil heavy metal pollution on food safety

Since crop production is influenced by soil quality and climate properties, the crop production showed high spatial heterogeneity. For example, the crops in the southeast of China are cultivated two or three times per year, while in northern China the crops are often cultivated only once a year. However, it is difficult to estimate the grain production per farmland area. Here we use the grain products in each province in 2012 to present the spatial variations of grain production. The proportion of affected grain production in each province accounting for the total grain production in China is calculated by:

$$RGPR_{m}=\frac{G_{m}}{\sum_{m=1}^{32}G_{m}}\times SPR_{m}$$ (3)

where *G*_*m*_ is the grain production in 2012 in province *m*, which could be obtained from the statistic book.

The proportion of grain production affected by heavy metal pollution in farmland soil on a national scale (*CGPR*) is calculated by:

$$CGPR=\sum_{m=1}^{32}RGPR_{m}$$ (4)

## Results and Discussions

### Heavy metal pollution in Chinese arable soil

The statistical information about the collected pollution rates of heavy metals is described in Table 1. The investigated areas for different heavy metals ranged from 954,500 km^2^ to 1,184,400 km^2^, accounting for a total area of arable land (1,353,850 km^2^) of 70.50% ~ 87.48% in China. These investigations covered 32 provincial-level administrative divisions save for Tibet Autonomous Region and Taiwan Province, indicating that the collected data covered most of China’s farmland soils. The notorious five poisonous heavy metals of Cd, Pb, Hg, As, and Cr gained much more attention than the other three metals, since the investigated soil samples were more than 116,000 points from over 355data records.

**Table 1:** Statistical information of the heavy metal pollution rate.

	Number of studies	Investigated area	Investigated samples	Pollution rate	Bulletin report*	Another review**
Cu	379	978619	104314	3.01	2.1	9.16
Pb	510	1113075	126385	0.96	1.5	0
Zn	328	954510	95213	2.09	0.9	1.53
Cd	481	1184393	127422	7.75	7	6.11
As	374	984386	119830	1.54	2.7	——
Ni	192	978356	82746	2.88	4.8	6.11
Cr	385	1099987	116261	0.60	1.1	0
Hg	355	1098668	116800	3.65	1.6	——

* This data was cited from the Bulletin of National Soil Pollution Survey, 2014

** This data was cited from the published paper by Niu et al. (2013).

Among the eight heavy metals, Cd showed the highest pollution rate of 7.75%, which was much higher than the other heavy metal pollution rates. This also denoted that Chinese farmlands were heavily polluted by Cd. The four metals of Zn, Cu, Ni, and Hg showed similar serious pollution in farmland soil, with the pollution rate from 2.09% to 3.65%. As, Pb and Cr pollution showed relatively low values, within the range of 0.60% to 1.54%. The characteristics of heavy metal pollution showed little difference between this study and the Bulletin. The Bulletin showed that Cd pollution in all kinds of soils was the worst with a pollution rate of 7%, Ni ranked the second most serious, then Cu, As, Hg, Pb, Cr and Zn were listed. These pollution rates in the Bulletin were for all kinds of land uses, but the Bulletin pointed out that the pollution rates of heavy metals in farmland soils were higher than in all other types of soils(Bulletin of National Soil Pollution Survey, 2014). In the study by Niu et al. (2013), the pollution rates for Cd, Cu, and Ni were higher than 6.0% while Zn, Pb and Cr had low pollution rates in China.

In the review study by Song et al. (2013), the pollution risk (the number of the studies with the higher averaged heavy metal concentrations than the reference II divided by the total number of collected studies) from Cd was the highest among the eight heavy metals, at 25.20%. This value was much higher than those found in our study and the Bulletin. The pollution risks from Ni, Hg, As and Pb were listed as the second serious, while pollution from Cu, Zn, and Cr showed relatively low risks. The big gaps in the heavy metal pollution rates between Song et al. (2013) and this study were mainly due to the different calculation methods.

Table 2 shows the frequency distribution of heavy metal pollution rates. It showed that the pollution rates of the eight heavy metals ranged from 0 to 100%. "0" indicates that the study area is not polluted by this metal, while 100% denotes the whole investigated area is polluted by this kind of metal. The pollution rates of Cr and Pb had the highest "clean rates", and more than 80% of the investigated regions had zero pollution rates. The clean areas for As, Zn, Cu, Ni, and Hg accounted for 50% to 75%, indicating the soils of more than half of the studies were totally clean of these five metals. The clean area from Cd pollution showed the lowest percentages among the eight heavy metals, with a value of 40.33%.

**Table 2:** Percentages of the data records with the pollution rates in different ranges.

Pollution ratio	0.00	0.01~10.00	10.01~30.01	30.01~50.00	50.01~99.99	100.00
Cu	60.69	16.62	8.71	1.06	6.33	6.60
Pb	82.16	7.84	3.53	2.75	2.16	1.57
Zn	63.72	14.94	7.62	3.66	4.88	5.18
Cd	40.33	18.50	10.40	7.90	9.77	13.10
As	72.73	16.84	5.61	1.87	1.34	1.60
Ni	56.77	23.96	10.94	2.60	2.60	3.13
Cr	84.94	11.95	2.08	0.52	0.26	0.26
Hg	58.59	18.87	9.30	4.23	6.48	2.54

The studies with the Cd pollution risks of 100% accounted for 13.10%, combining the percentages of "clean soil" and the sample-number-weighted mean of pollution rate, showing that Chinese arable soil faced high risk of Cd pollution. Moreover, the percentages of the studies with 100% pollution rates for Cu, Zn, and Ni were 6.60%, 5.18% and 3.13%. This denoted that although the general pollution rates for the three metals were not high, the pollution in some local areas was serious. Most of the investigated areas with high pollution rates were located in mining and smelting areas or in sewage irrigation areas[16–18].

Correlation measures the linear relationship between the pollution rates of soil heavy metal concentrations. The Pearson correlation coefficients and their significance levels (p < 0.01) between all these rates are presented in Table 3. The results showed that the eight pollution rates of the heavy metals correlated with each other, indicating that they might share common origins. Particularly, Cu had higher than 0.50 correlation coefficients with Pb, Zn, Cd, and As; Pb had high correlation coefficients with Cu, Zn, and As; the pollution rate of Zn showed high close relationship to the other 7 heavy metals; Cd had higher correlation coefficients with Cu, Zn, and As higher than 0.5; As had high correlation with Cu, Pb, Zn, Cd and Cr; Cr showed high correlation only with Zn and Pb; Hg only had high correlation with Zn and Cu; Ni had high correlation coefficients with Zn, Cd, and As.

**Table 3:** Pearson correlation coefficients between pollution rates of heavy metals in arable soil.

	Cu	Pb	Zn	Cd	As	Cr	Ni	Hg
Cu	——	0.59*	0.66*	0.58*	0.54*	0.47*	0.31*	0.48*
Pb		——	0.70*	0.41*	0.55*	0.36*	0.43*	0.37*
Zn			——	0.58*	0.45*	0.51*	0.46*	0.51*
Cd				——	0.50*	0.46*	0.17*	0.49*
As					——	0.48*	0.39*	0.20*
Cr						——	0.39*	0.25*
Ni							——	0.20*
Hg								——

* Levels of significance: p < 0.01.

### The potential human sources for heavy metals

The variations of heavy metals in farmland soils have been influenced by parent material and soil properties, as well as by human activities, such as industrial production, traffic, fertilizer addition, and irrigation. To find out the potential human sources of heavy metals in Chinese farmland soil, the heavy metal pollution rates in the 6 land uses were separately calculated.

#### Non-ferrous metal mining and smelting activities

The non-ferrous metal mining and smelting activities discharge large amounts of wastewater, waste gas and solid waste into the environment (Zhang et al., 2013). Thus the heavy metals in these wastes could enter into soil through atmospheric diffusion and surface runoff flushing or weathering [19,20]. Table 4 shows that different non-ferrous metal mining and smelting areas have different effects on the heavy metal pollutions. For example, Cu mining and smelting activities caused serious Cd, Cu and Hg pollution, with the pollution rates higher than 45%. Au mining and smelting activities leaded to obviously heavier Hg pollutions in soil than the other 7 kinds of heavy metals. The activities of Hg mining and smelting caused serious Cu and Hg pollution—higher than 70%, and also caused one third of Cd pollution. In Mo mining and smelting areas, Cd and Hg pollutions were very serious. In Mn and W mining and smelting areas, the soil was heavily deteriorated by Cu and Cd, and Pb pollution was also serious in Mn mining areas. Pb/Zn, Sb, and Sn mining and smelting activities could introduce large amounts of almost all of the eight kinds of heavy metals into soil, and caused serious Cu, Zn, Cd, Hg, and Ni pollution.

**Table 4:** The averages of heavy metal pollution rates in the non-ferrous mining and smelting areas (%).

Non-ferrous mining and smelting activities	Cu	Pb	Zn	Cd	As	Ni	Cr	Hg
Cu	63.64	0.00	12.12	83.67	0.00	0.00	0.00	45.74
Au	9.06	12.47	3.33	2.25	0.75	—	0.00	45.07
Hg	100.00	25.00	—	33.33	—	—	—	73.58
Mn	70.00	70.00	17.36	95.00	—	—	15.00	—
Mo	11.67	3.33	16.67	100.00	8.33	13.33	10.00	95.00
Pb/zn	57.61	49.64	72.23	74.43	29.42	44.60	9.28	68.51
Sb	82.35	100.00	88.24	100.00	94.12	100.00	100.00	100.00
Sn	100.00	14.04	75.00	21.05	2.22	50.00	—	—
W	95.00	20.00	30.00	100.00	100.00	—	—	—
Fe	3.40	0		0	0.85		0	0
Total	41.42	31.01	49.04	63.20	18.38	35.39	5.22	55.05

The non-ferrous metal mining and smelting activities introduced serious soil heavy metal pollution. Particularly, the Cd pollution rate was the highest with the value of 63.20%, and the pollution rates for Hg, Zn, and Cu were more than 40%, since almost all of the non-ferrous mining and smelting activities could cause serious Cd, Cu, Hg and Zn pollution. The pollution rates of Pb and Ni were about 35%, mainly due to the high pollution rates introduced by Sb, Sn and Pb/Zn mining and smelting activities. The pollution rate of As and Cr were listed as the lowest, mainly due to the Sb mining and smelting activities.

#### Non-metallic mining and smelting activities

Table 5 shows the heavy metal pollution rates in non-metallic mining and smelting areas. The activities of coal exploration caused high Hg and Cd pollution in soil, with the pollution rates higher than 20%. Coal mining also leaded to more than 10% pollution by Cu and Cr, about 7% pollution by Pb and Zn, and a little pollution by As and Ni. The realgar mining activities introduced a large amount of As into soil, but no other element pollutions were reported. In rare earth mining areas, the pollution rates of Cd, Cu, Hg and Pb showed as obviously higher than the other heavy metals. Cd pollution was the most serious in oil exploration areas.

**Table 5:** The averages of heavy metal pollution rates in the non-metallic mining and smelting areas (%).

Non-metallic mining and smelting activities	Cu	Pb	Zn	Cd	As	Ni	Cr	Hg
Coal exploration	13.80	6.69	7.35	20.14	1.03	1.45	10.66	26.27
Realgar ore	—	—	—	—	53.90	—	—	—
Rare earth mining	66.67	42.97	0.00	100.00	2.86	0.00	0.00	66.00
Oil exploration	0.00	0.00	0.00	96.63	0.00	0.00	0.00	0.00
Total	12.43	10.57	6.70	41.46	3.37	1.11	8.40	37.23

Compared to the heavy metal pollutions in non-ferrous mining and smelting areas, the pollution rates were lower in non-metallic mining areas. Among the eight heavy metal pollutions, Cd and Hg had high pollution rates, at about 40%. The Cu, Pb and Cr pollutions were listed as follows. The pollution rates for the other heavy metals, including Zn, As and Ni, showed relative low values.

#### Industry activities

We collected 8 data records on heavy metal pollution rates around industry plants. The pollution rates ranking from high to low were 43.99%, 37.21%, 35.17%, 6.50%, 7.69%, 0.64%, 0, and 0, for Cd, Ni, Cu, Hg, Zn, As, Pb and Cr, respectively. Cd had the highest pollution rates among the eight heavy metals, since cement and leather production introduced a large amount of Cd into soil[21,22]. Moreover, the cement production also caused heavier Cu pollution in soil, because the emitted gases contained a large amount of Cu[21]. The petrochemical complex leaded to a high Hg, Zn, Cu, and Cd pollution[23]. Here it should be noted that the heavy metal pollution in the reclaimed tidal flat soil showed as very serious, as high amounts of heavy metals would accumulated in the soil when wastewater ran into the sea[24].

#### Irrigation activities

Among the heavy metal pollutions in irrigation soils, Cr showed the lowest pollution rate of 0, indicating the irrigation water is clear from Cr pollution. The pollution rates of Cd were18.67%, and Pb, As, Ni, Hg pollution rates were listed as follows, with the value of 10.41%, 8.70%, 7.87%, and 6.18%. Zn and Cu showed relative lower pollution rates of 3.27% and 2.27%.

The Cu, Pb, and Hg pollution rates ranged from 0 to 62.50%, 75.76%, and 76.00%, respectively; while the Zn, Cd, As, and Ni ranged from 0 to 100%. This demonstrated that heavy metal pollutions varied greatly in irrigated areas, mainly due to the quality of the irrigation water. If low heavy metal contents were in the water for irrigation, the soil heavy metal pollution rates were low. If the irrigated water contained high heavy metal contents, the soils would be seriously polluted by heavy metals. Besides the water quality of the reclaimed water, the pollution in arable soil is also determined by irrigation rate, soil properties, crop usage and the irrigation period[25].

#### Urban development

Urban areas are the most densely populated regions with strong industrial and economic activities, which might greatly affect the soil characteristics in urban and sub-urban area[26,27]. The generation of municipal solid waste in China has increased at an average annual rate of about 9%, and only 2.1% of generated wastes was processed[10]. The soil in urban areas was most seriously polluted by Cd, with a pollution rate of 26.99%, indicating urban development and some industry activities introduced a large amount of Cd into soil. The soil polluted by Hg and Cu was also of concern, with pollution rates of 17.44% and 12.99%, respectively. Cr and Pb showed the low pollution rates among the eight heavy metals, with values of 0.35% and 1.24%. The other three metal pollution rates ranged between 4.75% and 8.35%.

#### Chemical fertilizer and insect inputs

If the soil was not affected by human activities, the heavy metal concentrations in most of remote areas would be far below their reference II limits. The exterior sources in remote areas mainly referred to normal agricultural practices of the application of animal manures or inorganic fertilizers, or pesticides (Atafaret al., 2010; Wu et al., 2013). Such applications may result in the increase of heavy metals, particularly Cd, Pb, and As [28]. The trace metal input of Cd, As, and Cr via fertilizers was close to or even higher than the metal input via atmospheric deposition in European agricultural soils [29]. In this study, soil Cd and Ni in remote areas had high pollution rates of 7.24% and 3.04%. More than 2% of samples were polluted by Cu and Hg, and more than 1% were polluted by As and Zn. The pollution rates of Pb and Cr were lower than 1%. Although the pollution rates in the remote areas did not show much serious pollution, it should be noted that fertilizer application is a nation-wide agricultural practice. The long-term usage of fertilizers might lead to high accumulations of heavy metal in the soil, and finally result in the arable soil being polluted.

**Fig 2:**
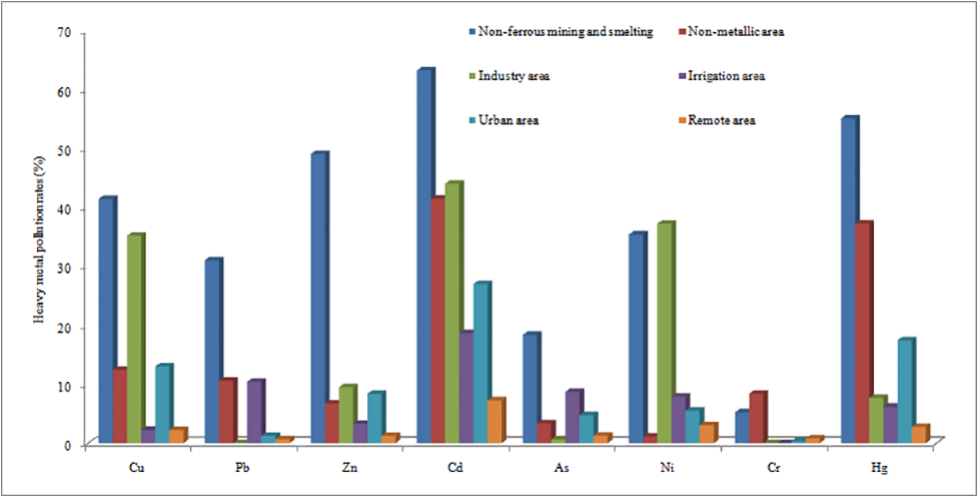
Heavy metal pollution rates from potential sources.

Comparing the sources of heavy metals in Chinese arable soil, the non-ferrous mining and smelting activities brought a large amount of heavy metals into soil, resulting in high heavy metal pollution rates (Fig 2). For the remote areas where chemical fertilizer and insecticide inputs were the main sources of heavy metals, the pollution rates showed the lowest. Urban areas had higher heavy metal pollution rates than remote areas. Non-metallic activities resulted in high Cd and Hg pollution rates. The heavy metal pollution by industry and irrigation activities was determined by the type of the production and the water quality used for irrigation. The soil pollution by heavy metals has aroused the public and government's concerns. The 12th Five-year Plan of Heavy metal Pollution Prevention and Control stated that the government should monitor the key industries including nonferrous metal mining, nonferrous metal smelting, lead storage battery production, leather production, chemical raw materials, and chemical products manufacturing, and 4452 key enterprises that emit heavy metals.

### Risk assessment of heavy metal pollution on food safety

Excessive accumulation of heavy metals in agriculture might result in soil contamination and elevate heavy metal uptake by crops, thus affecting food quality and safety[30]. Since the pollution rates of the eight heavy metals often showed different even in one place, the maximum pollution rate should be considered to assess their risks on food safety. About 10.18% of the investigated soil samples exceeded the reference II values, indicating that this arable land was not safe enough for crop planting. Among the 389 data records with polluted heavy metals, Cd contributed 47.87% of the maximum pollution rates, and then Hg, Cu, and Ni contributed 15.17%, 12.34%, and 9.48%. Zn, As, Pb, and Cr hadvalues lower than 7%. Form this point of view, the farmland soil were heavily polluted by Cd, Hg, Cu, and Ni, which together contributed about 84.85% of the total heavy metal pollution.

**Fig 3:**
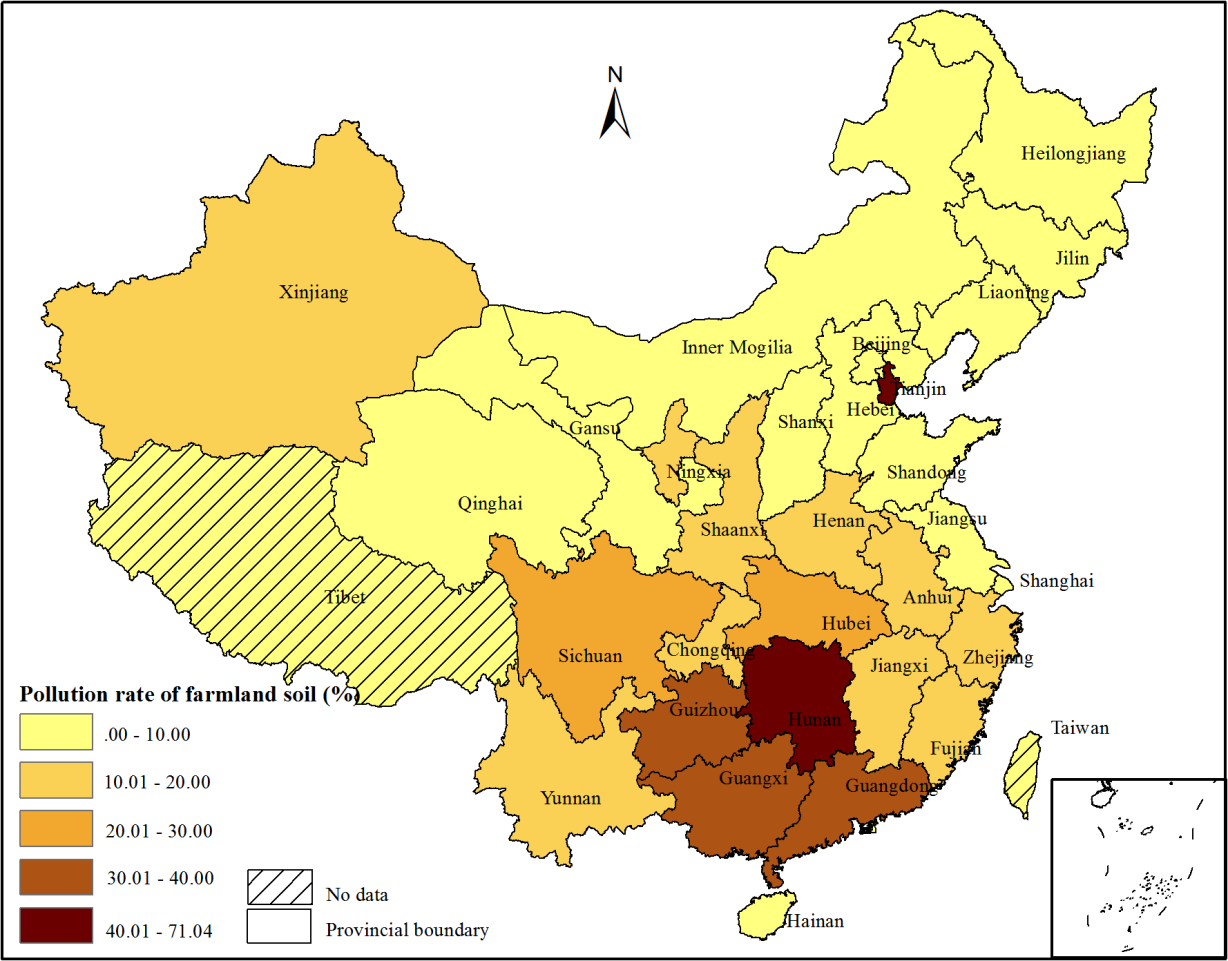
Pollution rate of farmland soil by heavy metals on a provincial scale

The pollution rate of farmland soil by heavy metals on a provincial scale is described in Fig 3. Tianjin had the highest pollution rates, at higher than 70%, mainly due to the high percentage of sewage irrigation in farmland soil. Hunan Province,Guangxi Zhuang Auto region, Guizhou Province, Guangdong Provincehad pollution rates of 55.93%, 36.25%, 38.75% and 30.80%, because there was a large amount of mining and smelting activities and chemical production plants. The pollution ratio by heavy metal in Sichuan, Hubei Province ranged from 20.00% to 30.00%. The provinces of Heilongjiang, Jilin, Liaoning, Hainan, Hebei, Neimeng, Qinghai, Shanxi, Beijing, Gansu, Shandong had relatively lower pollution ratios than 10.00%, while the remaining provinces were in the range of 10.01~20.00%.

**Fig 4:**
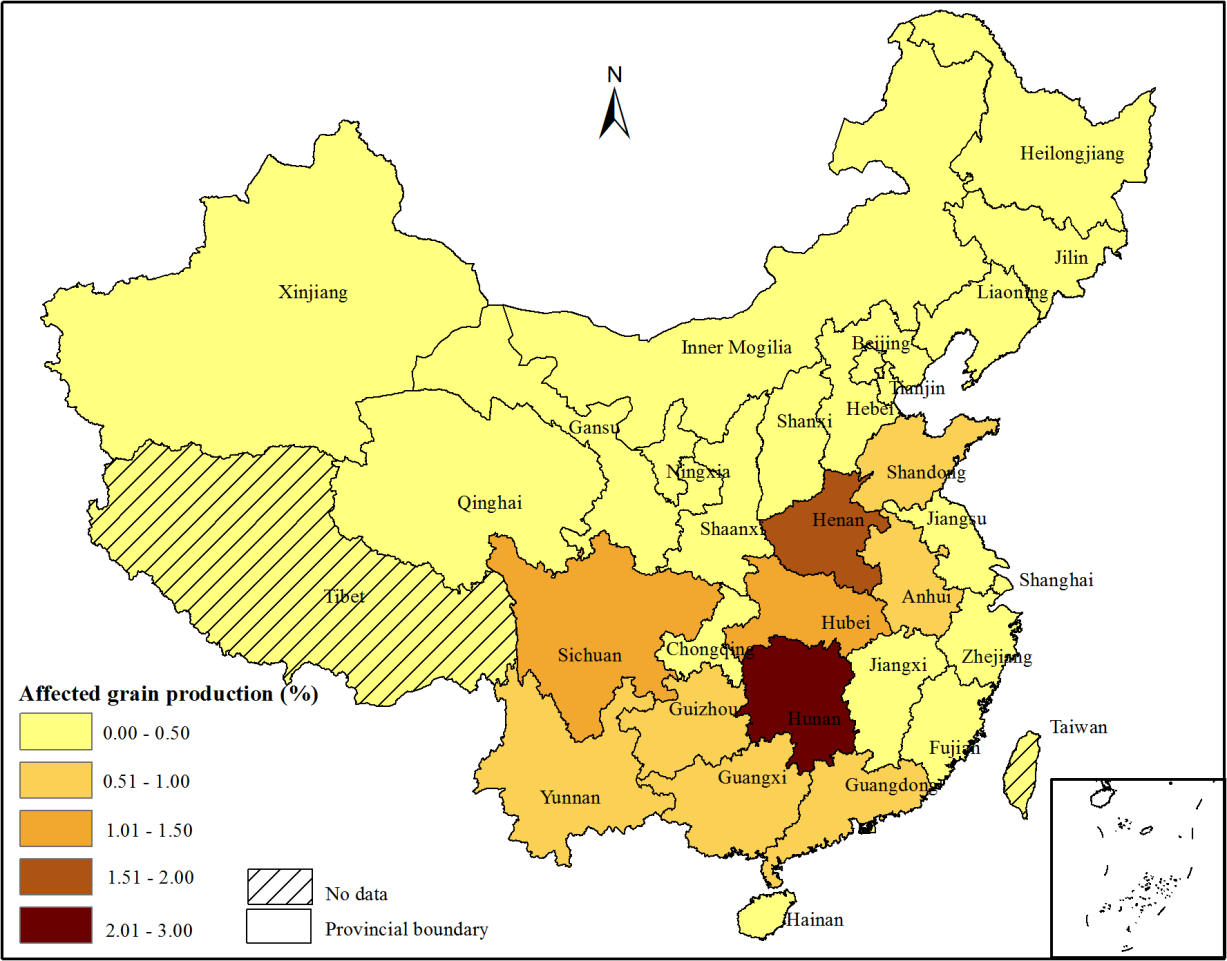
Percentages of affected grain production in each province accounting for the total grain production in China.

The proportion of affected grain production in each province accounting for the total grain production in China is described in Fig 4. Combing the crop production and the heavy metal pollution ratios in the 32 provinces, 13.86% of the grain production was affected by heavy metal pollution. This ratio was higher than the pollution rate of arable soil (10.18%). The reason might be that the areas polluted by heavy metal were mainly distributed in southern China, where there was high grain production per arable land due to the high temperature and numerous water sources for crop growth. Here, we should note that the affected grain production (13.86%) might refer to the decrease of the quality or quantity of grain production, but it did not mean that this percentage of grain production was polluted by heavy metals.

The highest grain loss was in Hunan Province, which accounted for 2.68% of the affected grain production in China, mainly due to the high soil pollution rate and grain production. Hunan Province is one of the top five producers of non-ferrous metals and it is also the largest rice producer in China[15]. The provinces of Henan, Sichuan, and Hubei accounted for1.51%, 1.28%, and 1.03% of the affected grain productions. The affected grain in Anhui, Guangdong, Guangxi, Shandong and Yunnan Province ranged from 0.5 to 1.0%. The remaining provinces accounted for lower than 0.5% of the affected grains in China.

The State Council has officially approved the 12th Five-Year Plan of Heavy Metal Pollution Prevention and Control and identified 14 provinces/autonomous regions, including Inner Mongolia, Jiangsu, Zhejiang, Jiangxi, Henan, Hubei, Hunan, Guangdong, Guangxi, Sichuan, Yunnan, Shaanxi, Gansu, and Qinghai, as key provinces/autonomous regions in heavy metal pollution management. The 14 most serious areas with farmland polluted by heavy metals occurred in Zhejiang, Anhui, Henan, Hubei, Hunan, Guangdong, Guangxi, Sichuan, Yunnan, Tianjin, Ningxia, Fujian, Guizhou, and Chongqing, among which are 6 provinces not found in the 14 provinces mentioned in the 12th Five-Year Plan. The 14 most serious areas with grain production polluted by heavy metals occurred in the provinces of Jiangsu, Zhejiang, Jiangxi, Henan, Hubei, Hunan, Guangdong, Guangxi, Sichuan, Yunnan, Anhui, Shandong, Guizhou, and Chongqing. The two pollution maps of arable soil and grains show similar spatial patterns (Figs 3 and 4). Most of the seriously polluted areas were located in the center and south of China. For the provinces of Shandong, Jiangsu and Jiangxi, the pollution rates by heavy metals were lower than 14%, but they listed in the top 14 provinces with polluted grains, mainly due to the high contribution of grain production in the three provinces to the total grain production in China. For the provinces of Ningxia, Tianjin, and Fujian, they had relatively high arable soil pollution rates without high grain pollution rates. This was mainly because these provinces were not high contributions to the total grain production of China.

### Uncertainties

First, the pollution situation in farmland soil changed year by year in China due to the fast economy and industry development. The data used in this study collected from 2005 to 2014 might bring uncertainties in the assessment of the situation by heavy metals. However, this problem seems inevitable because it is not easy to investigate the large area (1,353,850 km^2^) of farmland throughout China in a short time.

Second, although we tried to collect much data on the heavy metal pollution, the investigated area accounted for a total area of arable land (1,353,850 km^2^) of 70.50% ~ 87.48%, which might not represent the whole situation of China.

Third, the soil samples were not uniformly distributed throughout China since the sampling method is not system designed. The uneven distribution of soil samples may impact the consistency of the evaluation on heavy metal pollution. Particularly, some studies aimed to investigate the human activity influences on the heavy metal concentrations in soils, which might leaded to high pollution rate when we assessed the pollution situation on a regional or national scale. Moreover, some provinces had relative small samples, which also brought uncertainties.

## Conclusions

This study reviewed 465 studies on heavy metal pollution rates in Chinese farmland soil. Cd was ranked as the highest polluting heavy metal with the ratio of 7.75%, Hg, Cu, Ni, and Zn were next in that order, while the remaining heavy metals were not serious causes of pollution. Cu, Pb, Zn, and As showed high correlation coefficients amongst each other, and Cd had a close correlation to Cu and Zn, indicating that these heavy metals might share some common sources. The human activities of mining and smelting, industry, irrigation by sewage, urban development, and fertilizer application released certain amounts of heavy metals into soil, which resulted in arable soil pollution. In total, about 10.18% arable soil was polluted by heavy metals, and 13.86% of grain production in China was affected by heavy metal pollution in farmland soil.

## Supporting Information

S1 Table The information of the collected data records in this study (XLSX)
